# Moistube irrigation fouling due to anaerobic filtered effluent (AF) and horizontal flow constructed wetland (HFCW) effluent

**DOI:** 10.1038/s41598-021-86737-7

**Published:** 2021-03-29

**Authors:** T. L. Dirwai, A. Senzanje, T. Mabhaudhi, C. A. Buckley

**Affiliations:** 1 Agricultural Engineering Department, School of Engineering, University of KwaZulu-Natal, P.Bag X01, Pietermaritzburg, South Africa; 2 VarMac Consulting Engineers, Scottsville, Pietermaritzburg, 3209 South Africa; 3 Center for Transformative Agricultural and Food Systems, School of Agricultural, Earth and Environmental Sciences, University of KwaZulu-Natal, P.Bag X01, Pietermaritzburg, South Africa; 4 Center for Water Resources Research, School of Agricultural, Earth and Environmental Sciences, University of KwaZulu-Natal, P. Bag X01, Pietermaritzburg, 3209 South Africa; 5 Water, Sanitation and Hygiene Research and Development Centre, Chemical Engineering, School of Engineering, University of KwaZulu-Natal, Durban, 4041 South Africa

**Keywords:** Environmental sciences, Environmental social sciences

## Abstract

The study assessed the suitability of two effluent types, namely anaerobic filtered (AF) and horizontal flow constructed wetland (HFCW) effluent for Moistube irrigation (MTI). Secondary to this, the study determined the plugging coefficients (α) on MTI for the respective effluents. The feed water was supplied from a raised tank (3.5 m), and mass-flow rates were recorded at 15 min intervals using an electronic balance. The effluent feed water concentrations and experimental room temperature (25 °C ± 1 °C) were continuously monitored and kept constant. Hermia’s models based on the ${\text{R}}^{2}$ coefficient was used to select the best fitting fouling mechanism model and, consequently, the plugging coefficients. In addition, microbial colony analysis and scanning electron microscopy (SEM) analysis was carried out to assess the composition of the deposited sediment (DS) and adhered bacterial film (ABF) onto the MTI lateral. The study revealed that MTI pore blocking was a complex phenomenon described by complete pore-blocking model (${\text{R}}^{2}$ ≥ 0.50). Discharge followed an exponential decay with early fouling observed on AF effluent because of a high concentration of total suspended solids (TSS) and dissolved organic matter (DOM). Discharge declined by 50% after 20 and 10 h of intermittent operation for AF and HFCW effluent, respectively. The α for each effluent (foulant) were $\alpha_{AF}$ = 0.07 and $\alpha_{ HFCW}$ = 0.05, respectively, for AF and HFCW. The microbial analysis revealed bacterial aggregation structures that contributed to pore blocking. SEM imaging revealed complete surface coverage by deposited sediment. It is concluded that water quality determines the operation life span of MTI, and the two effluents promote accelerated MTI pore fouling or blocking. Continuous use without flushing the MTI will promote membrane degradation and reduced discharge efficiency. Additional filtration can potentially mitigate the membrane degradation process.

## Introduction

Agriculture consumes approximately 70% of the global blue water^1^. This exacerbates water scarcity in the wake of erratic rainfall fuelled by climate variability and change. Domestic wastewater can be a suitable alternative for irrigated agriculture. Wastewater is defined as water that has gone through anthropological change^2^. Wastewater treatment plants such as the decentralised wastewater treatment system (DEWATS) produce different types of effluents; for example, there is anaerobic filtered (AF) effluent and horizontal flow constructed wetland (HFCW) effluent. AF and HFCW effluents have varying degrees of microbial activity, total suspended solids (TSS) and total dissolved solids (TDS). AF is obtained after the effluent passes through the anaerobic baffled reactor (ABR) chamber, removing suspended solids and biodegradable organic material (BOM). The AF chamber further removes additional suspended solids, colloidal solids and further reduces BOM. For tertiary filtration, a combination of anaerobic filtration and constructed wetlands (CWs) filtration is applied. For example, the HFCW effluent is obtained by passing the AF effluent through a vertical flow constructed wetland (VFCW) which consists of planted gravel filters that aid in further filtration and removal of pathogens. The horizontal flow constructed wetland removes additional pathogens and suspended solids^3^. CWs are considered tertiary wastewater treatment mechanisms, and they significantly process wastewater for reuse^4^.

Although wastewater relieves pressure on freshwater bodies, the treated wastewater from a decentralised wastewater treatment system (DEWATS) contains heavy metals, pathogen and high nitrate concentrations that can contaminate the environment^5–7^. A combination of wastewater usage and water use efficient irrigation technologies such as Moistube irrigation (MTI) can potentially relieve the pressure on freshwater bodies and improve water use efficiency for crop production. According to Trooien et al.^8^, potential benefits such as replenishing phosphorous in the soil, minimal human contact, and improved nutrient control, to mention a few, are derived from using wastewater from animal lagoons. Also, micro-irrigation systems offer an opportunity to effectively control pollution by wastewater and simultaneously promoting agricultural production^9^.

MTI is a new technology that uses a semi-permeable membrane (SPM) to emit water continuously in response to the soil matric potential and the applied pressure. The SPM membrane is made from inorganic filler material mixed with surfactant ethylene oxide/oxirane (A EO-7) aliphatic alcohol polyethenoxy (7). The filler material facilitates the carboxymethylation reaction of the aliphatic alcohol polyethenoxy^10^. The SPM is characterised by nano-pores uniformly and densely distributed for maximised irrigation uniformity^11,12^. MTI is a low-pressure continuous irrigation method whose discharge is controlled by soil matric potential. The inner membrane closely simulating the vascular plant tissue and uses the soil-moisture gradient for advection^13^, and it assumes a line source infiltration mechanism during irrigation^11^. Table 1 summarises the membrane properties. The technology optimises irrigation field water use efficiency (fWUE) since it utilises on-demand water application^12,14^.

**Table 1:** 3rd generation Moistube membrane properties.

Property	Information
Material	Polymeric
Thickness (${\text{mm}}$)	1.1
Inside/outside diameter (${\text{mm)}}$	15.87/17.28
Area (m^2^ m^−1^ length)	0.1043
Pore size (${\text{nm}}$)	500 (average)
Nominal discharge (L h^−1^ m^−1^ length)	0.489

When used in irrigation, depending on irrigation technology, wastewater accelerates emitter clogging and fouling. Emitter fouling and clogging can be caused by physical, chemical and biological processes and components or particles^15,16^. Fouling is characterised by four elementary phenomena: deposition, re-entrainment or re-suspension, agglomeration and clogging^17^. Clogging can be classified as “later stages” of fouling, leading to blockage^17^. Membrane fouling is a process whereby fine soluble particles deposit on the surface of an SPM, facilitating pore narrowing and subsequently pore blocking. MTI, as like other membranes, is susceptible to membrane fouling.

A study by Bucks, Nakayama^15^ revealed how wastewater increased the coefficient of variation of micro-irrigation emitters. Puig-Bargués, Arbat^18^ investigated the effects of effluents on drip irrigation kits. They found that drip-kits exposed to secondary treated effluent clogged faster than those exposed to wastewater that underwent tertiary treatment. Li et al.^19^ investigated biofilms' formation around drip emitters and conclusively recommended prescribed frequent lateral flushings to remove bio-films that accumulate from sewage water. Another study by Song et al.^20^ tested various chlorination techniques to eliminate bio-clogging caused by reclaimed water. Kanda et al.^16^ revealed that total suspended solids (TSS) had a significant effect on MTI clogging as compared to total dissolved solids (TDS).

Technology has facilitated the means to perform microscopy analysis on irrigation emitters. Emitter clogging has over the years been analysed by high-resolution microscopy methods such as scanning electron microscopy (SEM), SEM- energy dispersive X-ray (EDX), transmission electron microscopy (TEM)^21^, scanning tunnelling microscopy (STM), and the atomic force microscopy (AFM)^22^. Energy-dispersive X-ray (EDX) is a powerful tool for breaking down sediment compounds into elemental composition^23^. Also, fractal analysis is made possible by grey-scale SEM imagery, thus allowing the analysis of sediment's physical make-up^22^.

DEWATS effluent undergoes multiple filtration stages; this provides an opportunity to irrigate with “least” expensive water. Wastewater promotes emitter clogging; thus, determining the plugging coefficients aids in understanding the MTI degradation process and the SPM’s capacity to perform irrigation effectively under varied effluent quality^24^. There is no empirical evidence on the performance and the fouling of MTI under selected wastewater effluents. Kanda et al.^16^ investigated the clogging effect of suspended solids and dissolved solids on MTI; however, the study did not quantify the extent of clogging due to wastewater. Furthermore, the study did not quantitatively determine the plugging coefficients. This study sought to address the following questions: (1) how different effluent type influences the irrigation performance of MTI and (2) what is the degree of clogging and the subsequent plugging coefficient associated with flux decline in the different effluents used for the study. The study offers significant insights into MTI degradation due to wastewater irrigation, subsequently providing information on operation and maintenance (O&M) of MTI when used wastewater is used for irrigation. The study further determined the plugging coefficients (α) for AF and HFCW effluents using MTI. The plugging coefficients can be adopted for the emitter discharge equation for MTI. The study hypothesised that wastewater effluent degraded MTI discharge capacity. A study by Bhattacharjee and Datta^25^ developed pore blocking models exclusively for gel formation, which limits its applicability on generating knowledge around MTI performance under permeate flux decline.

## Fundamentals of fouling

Fouling diminishes a membrane’s permeability^26^. The model formulated by Hermia^27^ forms the basis of other modified fouling models^28–31^, defined various membrane clogging stages as standard blocking, intermediate blocking, complete blocking and cake formation and characterised them according to Eq. (1):

1 $$ \frac{{d^{2} t}}{{dV^{2} }} = \left( {\frac{dt}{{dV}}} \right)^{n} = \alpha \left( \frac{1}{J} \right)^{n} $$

where $t$ is the filtration time (${\text{s}}$), $V$ is the filtrate volume per unit area (${\text{m}}^{3} \,{\text{m}}^{ - 2}$), $J$ is the filtration velocity (permeate flux) (${\text{m}}\,{\text{min}}^{ - 1}$), $\alpha$ is the plugging coefficient and, $n$ is the constant of proportionality for constant pressure filtration of a Newtonian fluid.

**Figure 1:**
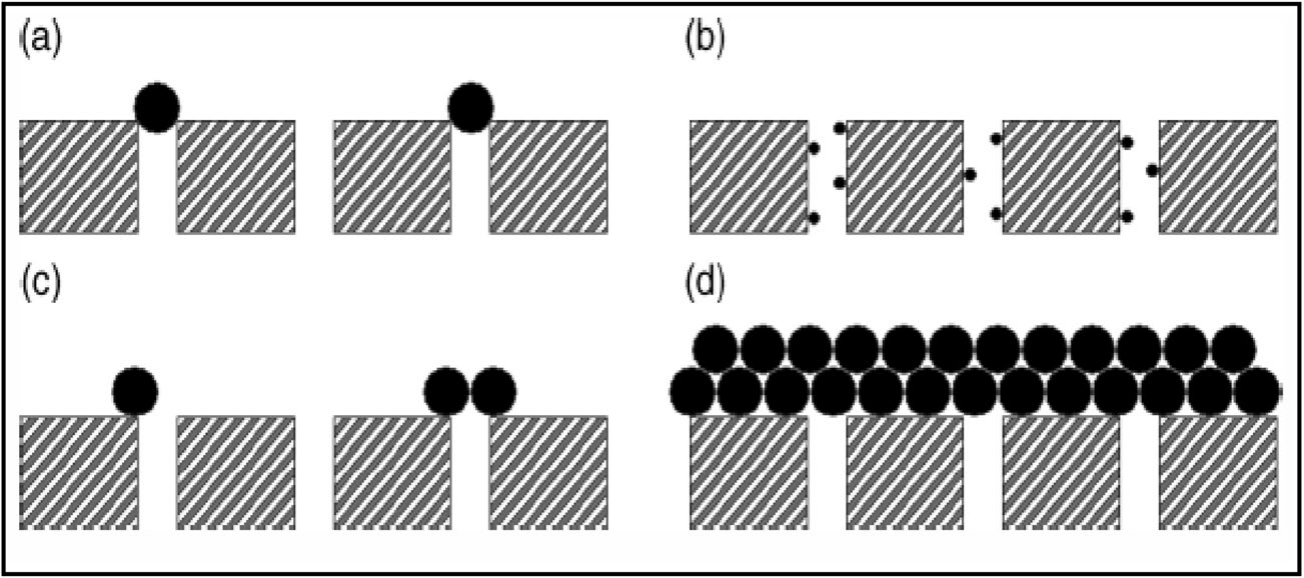
The various types of membrane fouling (**a**) complete pore blocking, (**b**) internal pore blocking, (**c**) partial pore blocking, and (**d**) cake formation after Field^33^.

Permeate flux decline is caused by pore blocking and cake deposition^32^. Field^33^ posited that membrane fouling occurs in stages, and there exist empirical functions that define each plugging or fouling stage (Fig. 1). For instance, the modified Eqs. (2)–(5) define standard, intermediate, complete fouling and cake formation, respectively^24,33^.

Standard blocking or pore narrowing occurs when the effluent soluble molecules have a lower diameter than the membrane pores^34^. The effluent molecules adhere to the emitting pores of the membrane and subsequently diminish its discharge. Standard blocking is characterised by Eq. (2) ^24^:

2 $$ J_{p} = \frac{{J_{o} }}{{(J_{o} + J_{o}^{0.5} \cdot \alpha_{s.} \cdot t)^{2} }} $$

where $J_{p}$ is the instantaneous permeate flux (${\text{m}}^{3} \,{\text{m}}^{ - 2} \,{\text{min}}^{ - 2}$), $J_{o}$ is the permeate flux (${\text{m}}^{3} \,{\text{m}}^{ - 2} \,{\text{min}}^{ - 2}$) at time $t = 0$, and $\alpha_{s}$ is the phenomenological standard blocking coefficient.

Corbatón-Báguena et al.^24^ defined intermediate blocking as a phenomenon that occurs when the effluent soluble molecules equal the membrane’s pore diameter. The corresponding function for the phenomenon is characterised by Eq. (3):

3 $$ J_{p} = \frac{{J_{o} \cdot J_{pss} \cdot \left( {e^{{\alpha_{i} \cdot J_{pss} \cdot t}} } \right)}}{{J_{p} + J_{o} \left( {e^{{\alpha_{i} \cdot J_{pss} \cdot t}} - 1} \right)}}, $$

where $J_{pss}$ is the permeate flux (${\text{m}}^{3} \,{\text{m}}^{ - 2} \,{\text{min}}^{ - 2}$) at steady-state flow, and $\alpha_{i}$ is the phenomenological intermediate blocking coefficient.

Complete blocking or pore sealing entails the complete blocking of the membrane pores^30^. The blocking mechanism is defined by Eq. (4)^24^.

4 $$ J_{p} = J_{pss} + \left( {J_{o} - J_{pss} } \right) \cdot e^{{ - \alpha_{c} \cdot t \cdot J_{o} }} , $$

where $\alpha_{c}$ is the phenomenological complete blocking coefficient.

Cake formation or gel layer formation occurs after complete blocking. The effluent solutes cannot pass through the membrane layer, and they form a cake layer. The mechanism has the general equation as in Eq. (5):

5 $$ t = \frac{1}{{\alpha_{gl} \cdot J_{pss}^{2} }} \cdot ln\left[ {\left( {\frac{{J_{p} }}{{J_{o} }} \cdot \frac{{J_{o} - J_{pss} }}{{J_{p} - J_{pss} }}} \right) - J_{pss} \left( {\frac{1}{{J_{p} }} - \frac{1}{{J_{o} }}} \right)} \right], $$

where $\alpha_{gl}$ is the phenomenological gel layer or cake formation coefficient.

The nano-pore size characteristics of MTI SPM have a cross-flow filtration effect on the AF and HFCW effluent; thus, the Hermia model was adopted since it characterises cross-flow filtration. Studies by Chang et al.^28^, Abdelrasoul et al.^35^, and Corbatón-Báguena et al.^24^, to mention a few, successfully adopted Hermia’s filtration model on various membranes.

Drip irrigation emitter discharge is described by Eq. (6), and when there is emitter clogging, the discharge equation is modified as in Eq. (7) ^36,37^:

6 $$ q_{e} = kH^{x} , $$

7 $$ q_{e} = \left( {1 - \alpha } \right)kH^{x} , $$

where *q*_*e*_ is the emitter discharge (L min^−1^), *k* is the emitter proportionality constant, *H* is the emitter operating pressure (m), and *x* is the emitter exponent and characterises the type of emitter.

According to Kanda et al.^16^, MTI discharge follows the general emitter discharge equations and is characterised by Eq. (8) ($R^{2} = 0.978$), and the presence of clogging reduces the discharge capacity by a factor $\alpha_{i}$ giving rise to Eq. (9):

8 $$ q = 0.1116h^{1.1948} , $$

9 $$ q = \left( {1 - \alpha_{i} } \right)0.1116h^{1.1948} , $$

where $q$ is the discharge, $h$ is the pressure head from the feed water supply, and $\alpha_{i}$ is the plugging coefficient.

## Materials and methods

### Study site and effluent characteristics

The laboratory experiment was carried out at the University of KwaZulu-Natal Ukulinga Research Farm in Pietermaritzburg, KwaZulu-Natal, South Africa (29° 39′ 44.8″ S 30° 24′ 18.2″ E). AF and HFCW effluents were used to determine the plugging coefficients. AF has a high microbial load whilst HFCW is produced after the secondary filtered AF passes through sub-surface filters. The effluent was obtained from a decentralised wastewater treatment system (DEWATS) in KwaMashu, Durban, South Africa (29° 45′ 49.0″ S 30° 58′ 34.6″ E). DEWATS is a modular water sanitation system consisting of settler, anaerobic baffled reactor (ABR) + anaerobic filter (AF) and planted gravel filters^38,39^. Other experiments such as microbial analysis and scanning electron microscopy (SEM) were done at the University of KwaZulu-Natal (Pietermaritzburg) plant pathology laboratory and microbiology and microscopy unit (MMU), respectively.

Musazura^5^ characterised the two types of effluents as per Table 2. The on-site laboratory analysed for $NH_{4}^{ + } - N,$ $NO_{3}^{ - } {-\!\!-}N$, and $PO_{4}^{3 - } - P$ using a NOVA 60 Merck Spectroquant (Merck Millipore, Germany) following standard methods for water and wastewater analysis^40^. According to Capra and Scicolone^41^, the tabled parameters (Table 2) can be used to evaluate emitter fouling exclusively.

**Table 2:** DEWATS effluent quality characterisation and the corresponding emitter clogging risks^42^.

Constituent	Concentration			Emitter clogging risk		
				Hazard rating		
	Units	AF	HFCW	Minor	Moderate	Severe
**Physical**						
Electrical conductivity	mS m^−1^	95	65			
pH		7.5	7.2		✓	
**Solids**						
Total suspended solids	mg L^−1^	579	575			✓
Total dissolved solids	mg L^−1^	476	543		✓	
**Nitrogen compounds**						
Ammonium	mg L^−1^	61	1			
Nitrates	mg L^−1^	0.1	4.1			
**Phosphorous**						
Ortho-phosphates	mg L^−1^	10.5	6.7			
**Bacteria**						
*E. coli* (CFU per 100 mL)		3.5 × 10^05^	5 × 10^02^		✓	✓

*CFU* colony forming units.

The above-listed characterised effluent can further be classified according to the severity of pore-blocking by the effluent's active foulants. Bucks et al.^42^ hazard rating scale was adopted to explain how each effluent's physical, chemical, and biological characteristics influenced pore narrowing and the subsequent pore blocking.

### Experimental set-up and procedure

**Figure 2:**
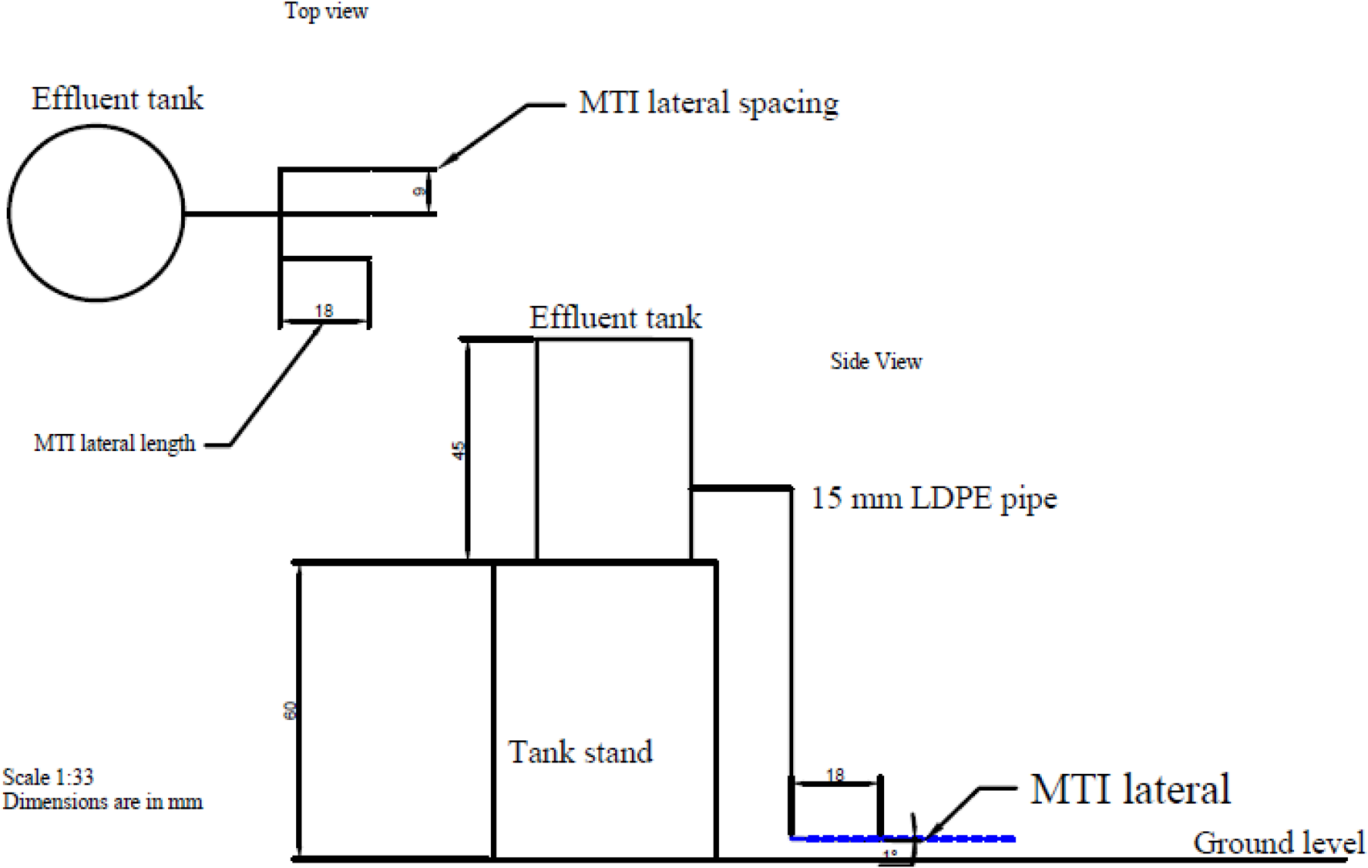
Experimental set-up.

The experiment equipment (Fig. 2) consisted of two 250 L tanks placed at a height of 3.5 m to facilitate enough head for fluid flow^16^. Each tank had a different effluent type. MTI lateral tubing of length 0.6 m were assembled in manifold arrangement and replicated three times. A 15 mm nylon end plug sealed each MTI lateral end. The pressure head was kept at 3.5 m; thus, there were minimal fluctuations in permeate flux velocity (0.49 l h^−1^ m^−1^ length). According to Kanda et al.^16^, there are no guidelines for manifold length, but standard practice dictates selecting a length that minimises frictional losses.

The lateral spacing was 0.3 m^16^, and the MTI laterals were placed in gutters laid at a gentle slope of 1% for ease of collection of the MTI discharge. The experiment was operated intermittently^18^ for approximately 60 h with start and end times of 08h30 and 16h00, culminating in 7.5 h day^−1^ for 8 consecutive days, respectively. No MTI flushing happened during the experimental period. The mass-flow rate collected in each gutter was recorded at 15-min intervals using a 1 kg full capacity electronic balance with a 1 g resolution.

The experiment was carried in a closed room where conditions were kept at room temperature (25 °C $\pm$ 1 °C) and atmospheric pressure to minimise evaporation effects. The effluent feed water quality was periodically tested for uniformity throughout the experiment using portable handheld TSS meter from HACH industries (TSS resolutions of 0.1 at 10–99.9 g L^−1^ and 1 at greater than 100 g L^−1^), and HI98129 combo tester for pH/EC/TDS/temperature from Hanna Industries (resolutions of 0.01 pH, 1 μS cm^−1^, 1 ppm of TDS, 0.1 °C). To avoid settling suspended solids, a low-head submersible pump was placed at the bottom of the tank for continuous mixing. Water levels were periodically checked at 5-min intervals using a graduated dip-meter. The effluent was periodically topped up to maintain the constant head.

### Method of analysis

#### Statistical analysis

R Studio^43^ was used for statistical analyses. The one-way ANOVA was applied to assess differences in discharges among laterals of the same treatment and discharge from the different wastewater treatments.

#### Mass flow rates

The mass flow rates were converted to volumetric discharge by multiplying the weight per unit time by the density $\left( {\rho_{w} } \right)$ of water. The permeate flux ($J_{o}$) was obtained from Eq. (10):

10 $$ J_{o} = \frac{Q}{A \cdot \Delta t}, $$

where $Q ({\text{m}}^{3} \,{\text{min}}^{ - 1} )$ is the discharge at time $\Delta t$ (${\text{min}}$), $A \left( {{\text{m}}^{2} } \right)$ is the membrane total surface area determined by Eq. (11), the surface area of a hollow cylinder:

11 $$ A = 2\pi l_{MTI} \left( {R + r} \right) + 2\pi \left( {R^{2} - r^{2} } \right), $$

where $l_{MTI}$ is the MTI lateral length (m), $R$ is the MTI external radius (m), $r$ is the MTI internal radius (m).

#### Relative discharge and degree of clogging

The other selected criteria or measures for assessing emitter clogging were relative discharge ($q_{rel}$) and time taken for discharge to reduce from 95 to 50%^44^ and the degree of clogging ($DC$)^45^. The relative discharge ($q_{rel}$) and $DC$ were calculated by Eqs. (12) and (13) as follows:

12 $$ q_{rel} = \frac{{q_{ini} - q_{t} }}{{q_{ini} }} \times 100, $$

13 $$ DC = \left( {1 - \frac{{q_{t} }}{{q_{ini} }}} \right) \times 100, $$

where $q_{t}$ is the instantaneous discharge at time $t$ (h), and $q_{ini}$ is the initial discharge (L h^−1^ m^−1^).

#### Bacterial quantity

**Figure 3:**
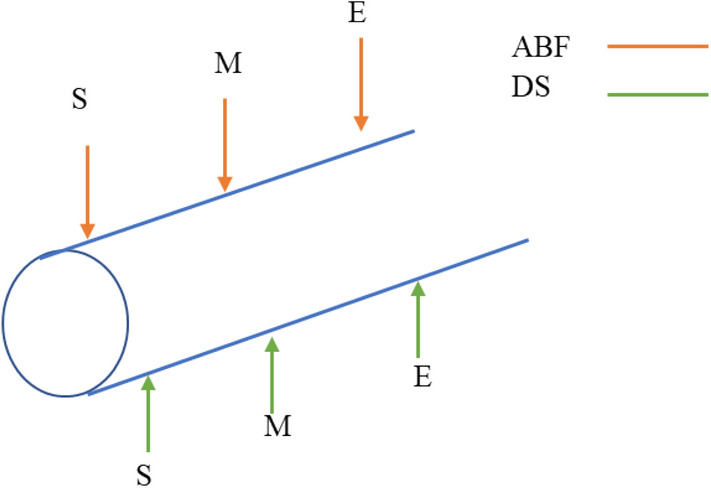
Sampled DS and ABF points along the MTI lateral for each respective effluent.

The study assessed the bacterial colonies that formed along the MTI lateral walls, hereafter referred to as adhered bacterial biofilm (ABF) and the bacteria that was deposited at the bottom section of the MTI lateral, hereafter referred to as deposited sediment (DS). The bacterial agar consisted of a mixture of 12 g Agar No.2 Bacteriological (Neogen Company, Heywood, Lancashire, UK) and 16 g of nutrient broth (Biolab Modderfontein, South Africa). The reagents were mixed to a solution of 1000 ml and stirred till boiling for 15 min. Considering that the irrigation intervals were intermittent, i.e., the laterals were allowed to drain during non-irrigating hours, hence the bacterial culture used catered for both aerobic and anaerobic bacteria. The culture mixture was then autoclaved at 150 °C for 1 h before being transferred to petri-dishes. The stock solution was obtained by cutting sections of the MTI laterals into 1 cm × 1 cm coupons. The coupons were allowed to soak for 72 h in de-ionised water. The sample to be tested was then diluted to 1 × 10^−5^ and 1 × 10^–6^ dilution from dispersed bacteria's stock solution. A 0.1 ml diluted solution was then applied to the medium and cultured in a 28–30 °C incubator for 48 h. A sterilised inoculating loop was dipped into each bacterial solution to collect bacteria from the dispersed bacteria sample and then streaked into each petri dish. The number of bacterial colonies was detected by the plate counting method^46^. For both the AF and HFCW effluents, all three laterals were sampled three times for DS and ABF. The laterals were sampled on three segments which were the start (S) of the lateral, mid-section (M) and the end section (E) of the lateral (Fig. 3).

#### Scanning electron microscopy (SEM) with energy dispersive X-ray (EDX)

For sediment analysis, the Scanning Electron Microscopy (SEM) was employed. SEM—energy dispersing X-ray (EDX) was also used to analyse the two sediments' elemental composition (ABF and DS). The sediment sample to be analysed was cut into small pieces measuring approximately 1 cm × 1 cm and mounted on a stub, and secured using insulated carbon double-sided tape. To facilitate conductivity, the samples were sputter-coated by gold using the Quorum Q150R ES machine. The viewing was done on a Zeiss EVO LS 15 machine with a resolution of up to 3072 × 2304 pixels. The samples were subjected to observational fractal analysis to assess the space occupied on the MTI inner surface by the irregular and potentially clogging particles^46^. For both effluents, material for DS and ABF sediment analysis was taken from each MTI lateral's mid-section.

## Results and discussion

**Figure 4:**
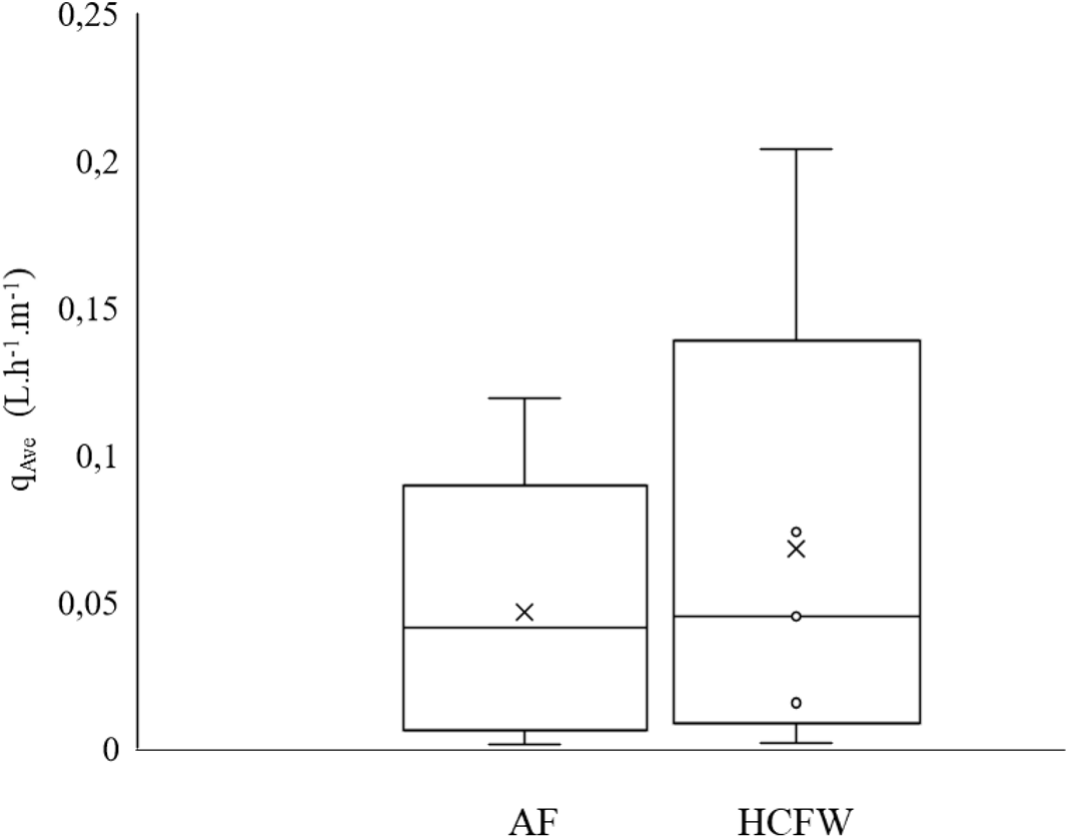
Box plot showing discharge variations between the two types of effluents.

A one-way ANOVA revealed no significant differences ($p > 0.05$) in discharges amongst the laterals for the respective effluents. The discharge from individual MTI laterals from AF and HFCW had approximately equal means and medians (Fig. 4), revealing a constant discharge from the respective laterals and replicates. The one-way ANOVA analysis, however, showed a significant difference ($p < 0.05$) in clogging due to AF and the HFCW effluent. This meant that effluent quality influenced the degree of clogging.

### Degree of clogging ($DC$) and relative discharge $q_{rel}$

**Figure 5:**
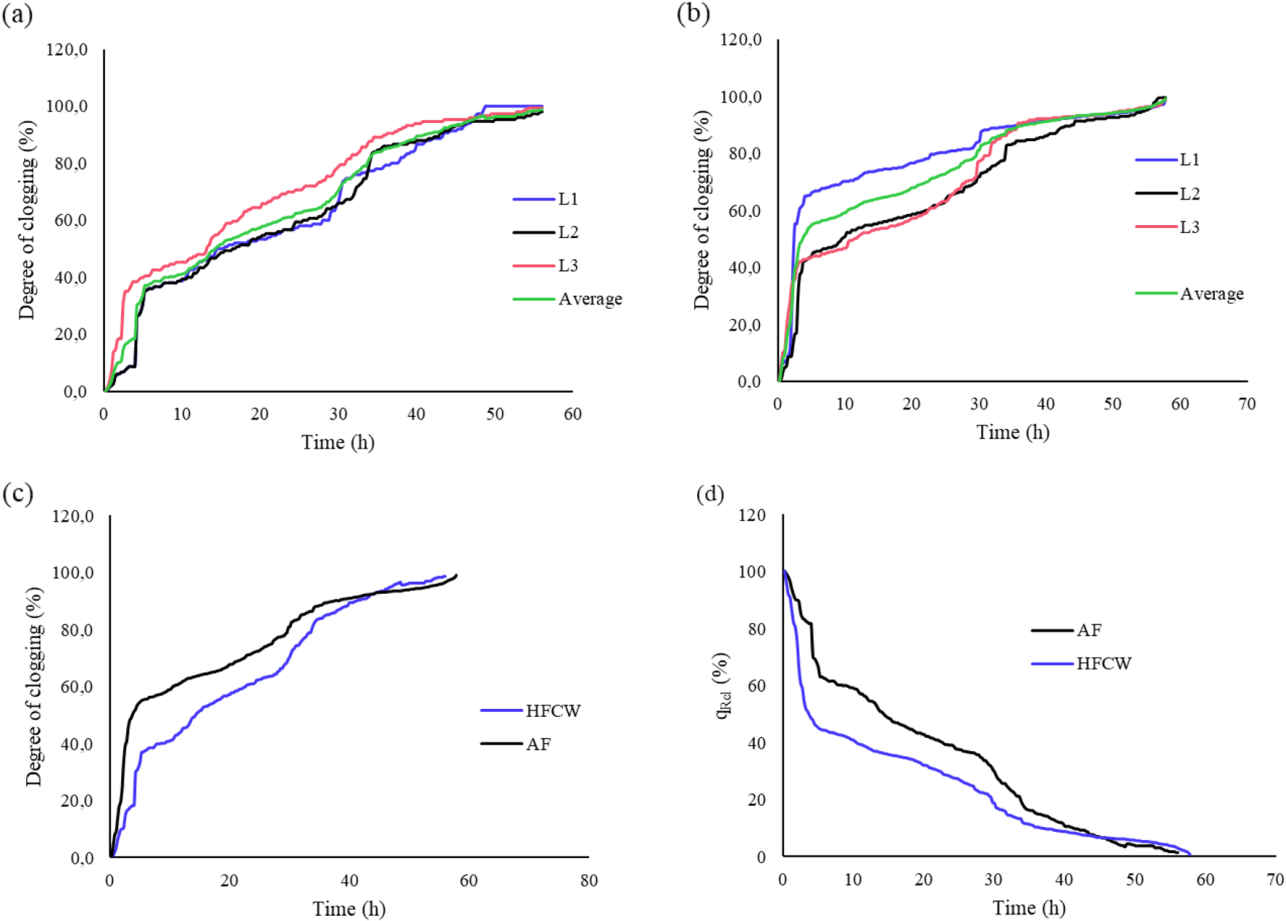
Degree of clogging ($DC$) for (**a**) AF effluent, (**b**) HFCW effluent, (**c**) $DC$ vs $q_{Ave}$ for AF and HFCW effluents, and (**d**) Relative discharge ($q_{rel}$) plots for AF and HFCW effluents.

The degree of clogging increased with operation time (Fig. 5a–c). AF presented a high clogging risk for the MTI laterals. AF had approximately 60% $DC$ from $t$ = 0 h to $t$ = 5 h, whilst HFCW had approximately 40% $DC$ over the same period. The phenomenon was potentially attributed to physical bore blocking caused by a high concentration of total suspended solids (TSS) in both effluents. A high TDS concentration (476–543 mg L^−1^) potentially contributed to the MTI nano-pores' constriction. A high TSS concentration in sewage sludge promotes complete pore blocking and subsequently cake formation^47^.

There was a significant difference in $q_{rel}$ between AF and HFCW effluent (Fig. 5d) ($p$ < 0.05). The significant drops in $q_{rel}$ during the beginning stages of the experiment were attributed to the presence of active and effective MTI pores as the SMP gradually wets; this concurs with the observations by Kanda et al.^16^. The time required for $q_{rel}$ to get to 50% ($T_{{q_{rel} }} \le 50\%$) under HFCW was low at 5 h as compared to AF at 15 h. This rapid decline for the HFCW effluent could be attributed to the high concentration of TSS in the effluent which according to the Bucks et al.^42^ hazard risk rating scale causes severe pore narrowing or clogging risk.

$$T_{{q_{rel} }} \le 10\%$$ for HFCW effluent was $$t$$ = 37 h and for AF effluent was $$t$$ = 41 h, after which both MTI discharges reached a steady state. Pore blocking for AF effluent occurred at about $$t $$ = 47.5 h with $$q_{rel}$$ of approximately 4%, whereas for HFCW, there was a steady decline with a seemingly asymptotic relative discharge of $$q_{rel} = 10\%$$ from $$t$$ = 51 h to $$t$$ = 56 h. The HFCW effluent $$q_{rel}$$ declined steadily again from 10% to approximately 8% from $$t$$ = 56 h to $$t$$ = 57.75 h.

### Permeate flux decline

**Figure 6:**
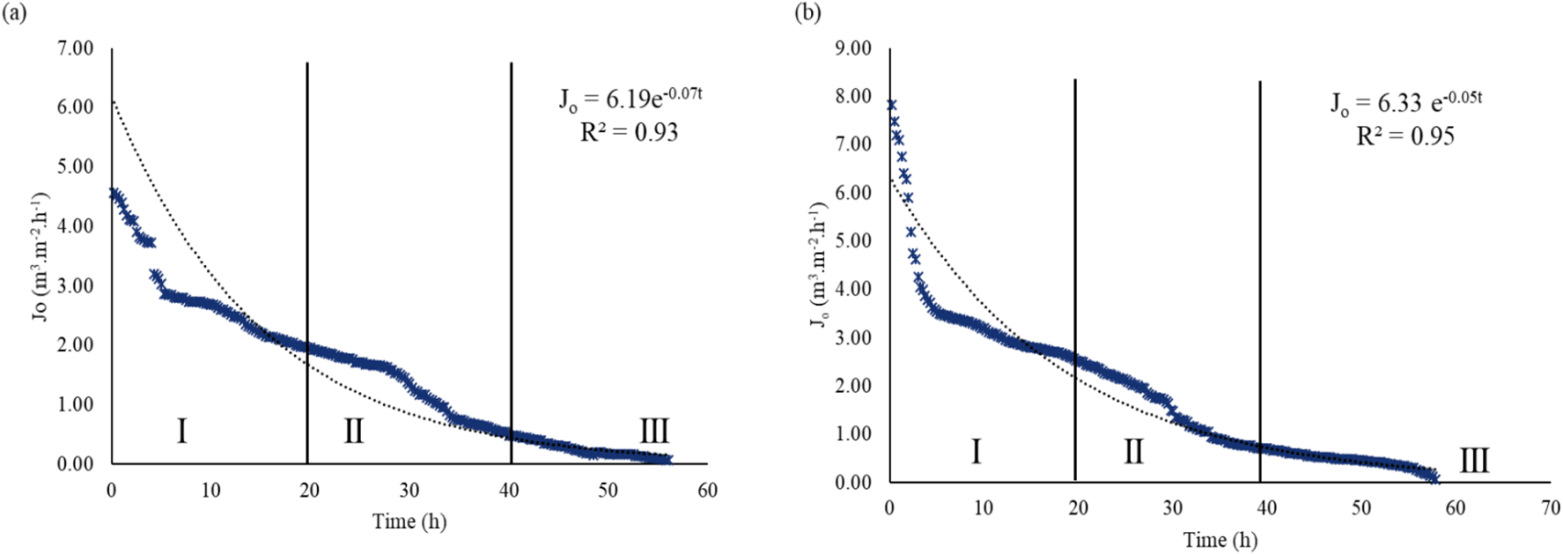
Flux vs time curve for (**a**) AF and (**b**) HFCW effluent.

Flux decline for AF and HCFW effluent followed an exponential decay function with $R^{2} = 0.93$ and $R^{2} = 0.95,$ respectively (Fig. 6). The flux decline exemplified a typical flux vs time curve wherein the first stage showed a rapid decline in flux (Stage I), followed by a protracted gradual flux decline (Stage II) and a steady-state flux decline (Stage III). The sharp decline in flux is reported in Lim and Bai^48^, where a hollow membrane used for micro-filtration of activated sludge wastewater showed a reduction in discharge over time. The flux–time data were modelled against the linear, logarithmic, polynomial and exponential functions, and the exponential function produced satisfactory results based on the respective $R^{2}$ values. Satisfactory R^2^ values range from $R^{2} \ge 0.50$^49,50^. Previous membrane clogging studies have modelled their experimental results against different functions and reported that the power function produced satisfactory $R^{2}$ values^48,51,52^. Similar to previous studies^48,51,52^, the selected power function encompassed the collective flux–time data points.

The various stages in membrane fouling under AF and HFCW effluents were characterized by the functional relationships as in Eqs. (14) and (15), respectively.

14 $$ J_{o} = 6.19e^{ - 0.07t} $$

15 $$ J_{o} = 6.33e^{ - 0.05t} $$

Maximum permeate flux was observed from $t$ = 0 h to $t$ = 10 h for both effluents because all MTI pores were available for discharging the effluents. The long-term gradual flux decline (II) was attributed to the high concentration of suspended solids. Extended MTI pore blockage (III) resulted in a steady-state permeate flux discharge for AF at $t$ = 45 h and $t$ = 50 h (approx.) for HFCW effluent.

The accelerated pore narrowing of AF effluent is attributed to the high concentration of TSS (49 mg L^−1^) compared to HFCW (TSS = 20 mg L^−1^) as well as to a high concentration of ortho-phosphates in AF effluent (10.5 mg L^−1^) and ammonium (61 mg L^−1^). Ortho-phosphates and nitrates can be classified as dissolved inorganic matter (DOM), and Tang et al.^53^ reported that DOM contributes 26–52% to membrane fouling. The findings concur with Capra and Scicolone^41^, whose study revealed a high TSS concentration and organic matter caused accelerated clogging of drip emitters. Effluent quality influences MTI fouling as dictated by the Bucks et al.^42^ hazard rating scale.

#### Plugging coefficients

**Figure 7:**
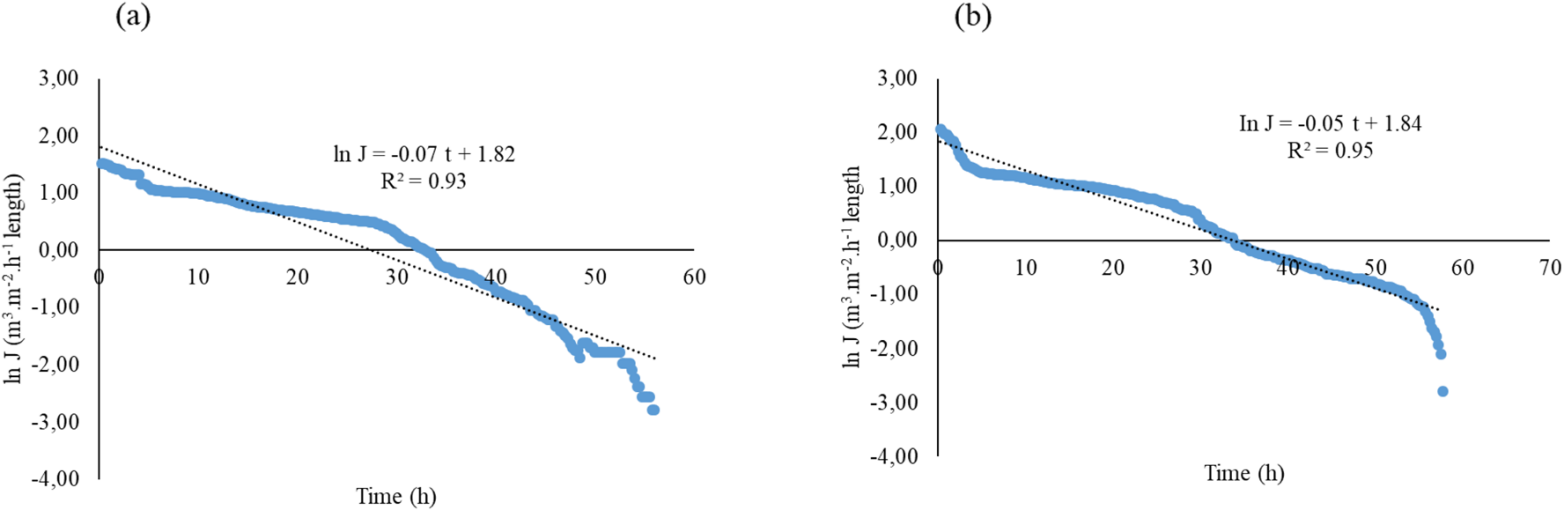
Linearised flux decline rate for (**a**) AF effluent and (**b**) HFCW effluent.

In practice, pore-blocking occurs in stages^32,54^. The linear relationship derived from the flux vs time curve yielded a functional relationship shown in Fig. 7. Effluent quality influences the plugging process. It is worth noting that pore-blocking occurs in stages; thus, membrane plugging complexity makes it difficult for one model to fit one plugging model.

The derived plugging coefficients from the respective effluents were; $\alpha_{AF}$ = 0.07 and $\alpha_{HFCW}$ = 0.05. The resultant superimposed MTI discharge function was then characterised as follows

16 $$ q_{AF} = \left( {0.93} \right)0.116h^{1.1948} , $$

17 $$ q_{HFCW} = \left( {0.95} \right)0.116h^{1.1948} . $$

The marginal difference in $\alpha$ under the two effluents permits for low ($t$ = 0 h to approx. $t$ = 60 h) intermittent MTI intervals. An intermittent drip irrigation experiment using urban wastewater by Capra and Scicolone^41^ that operated for 4–6 h for 60 h revealed that emitters with low discharge rates clogged faster than high discharge rate emitters. Continued MTI usage over $t$ = 60 h resulted in a 95% decline in flux.

### Bacterial activity

**Figure 8:**
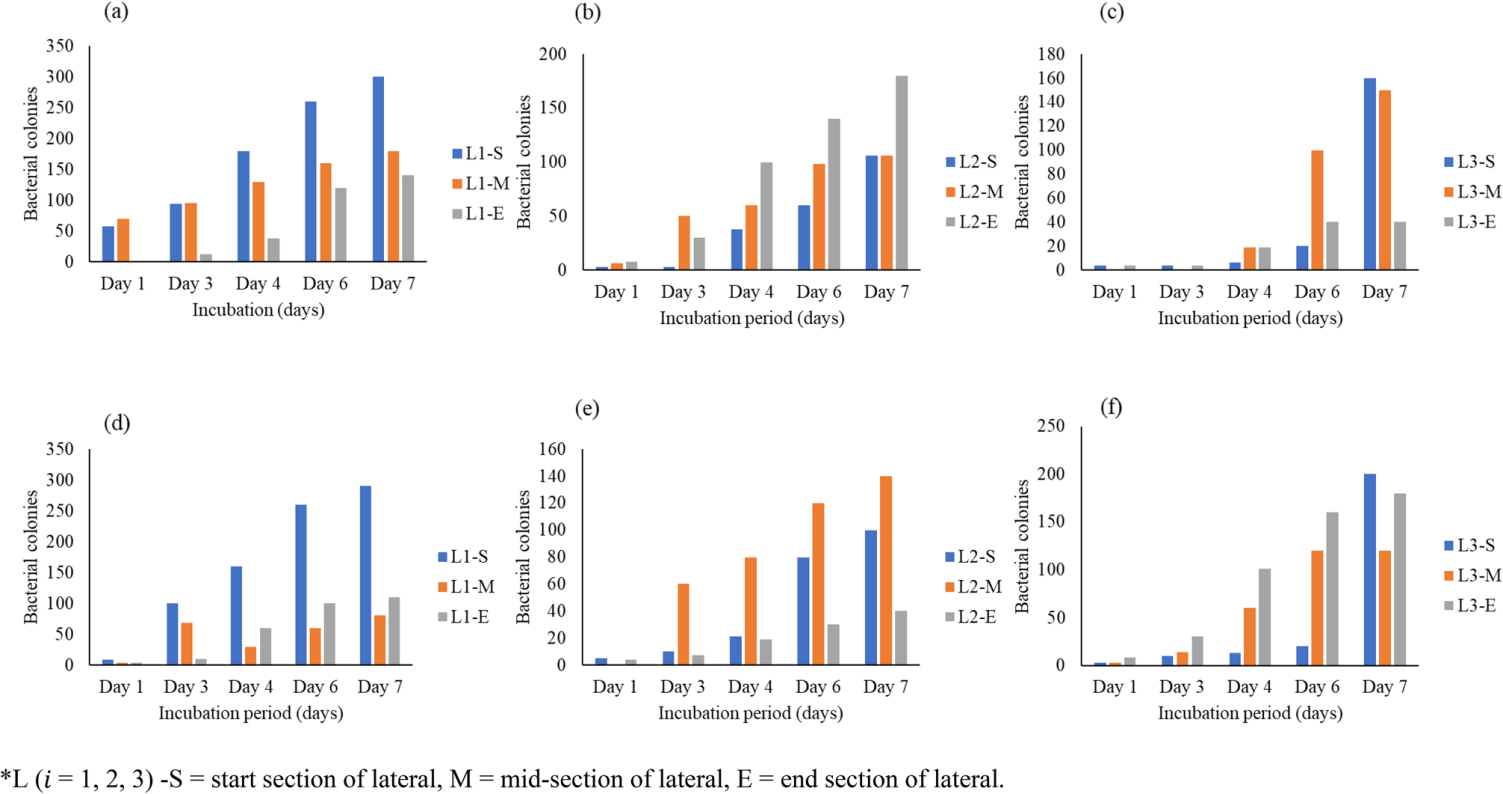
Bacterial colony growth for deposited sediment (DS) (**a**) colony growth under AF for lateral 1 (L1), (**b**) lateral 2 (L2), and (**c**) lateral 3 (L3). Bacterial growth on adhered bacterial biofilm (ABF) for (**a**) L1, (**b**) L2, and (**c**) L3. *L ($i$ = 1, 2, 3) *S *start section of lateral, *M* mid-section of lateral, *E* end section of lateral.

**Figure 9:**
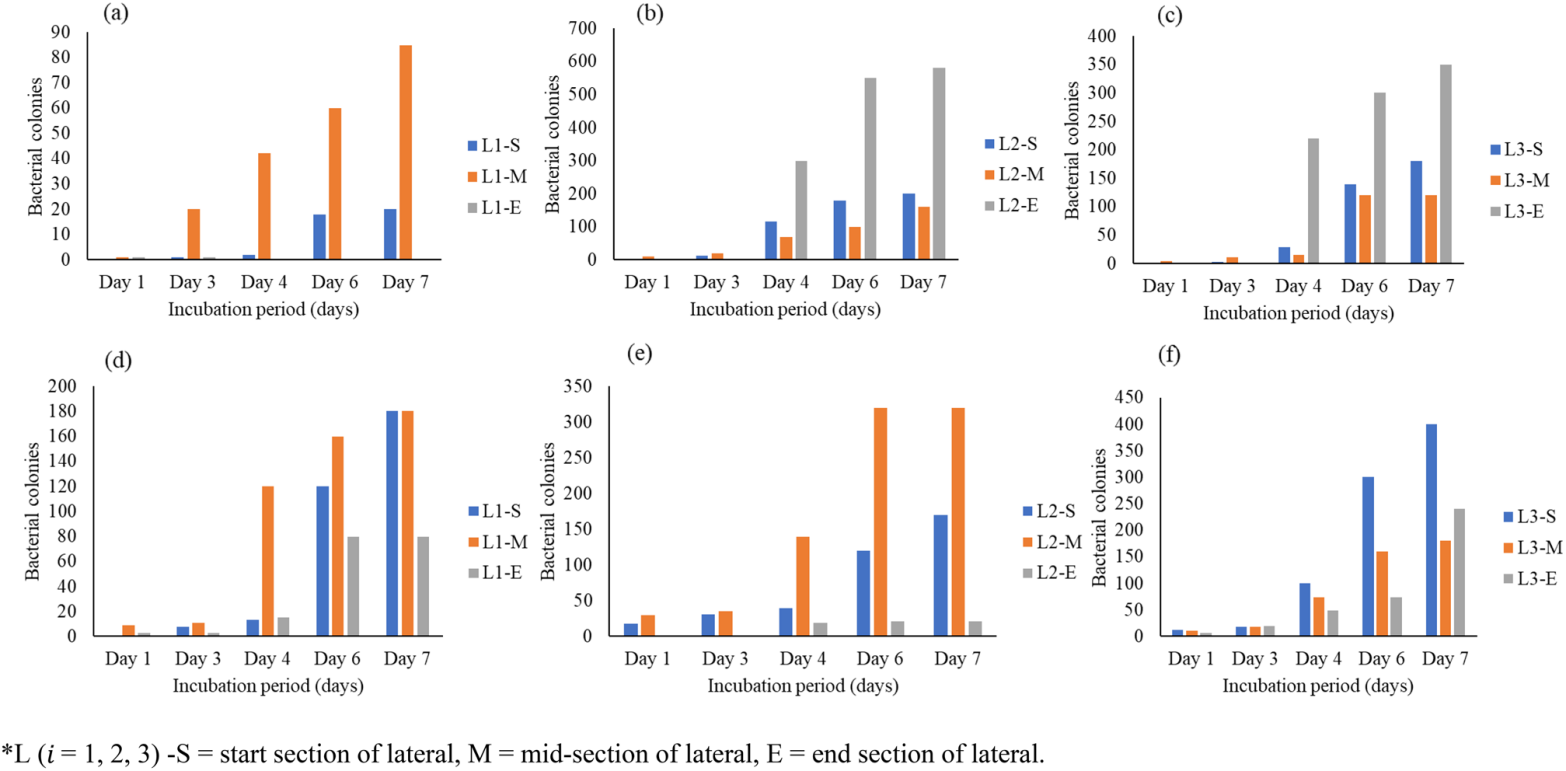
Bacterial colony growth for deposited sediment (DS) under HFCW effluent (**a**) for lateral 1 (L1), (**b**) lateral 2 (L2), and (**c**) lateral 3 (L3). Bacterial growth on adhered bacterial biofilm (ABF) for (**a**) L1, (**b**) L2, and (**c**) L3. *L ($$i $$ = 1, 2, 3) *S *start section of lateral, *M* mid-section of lateral, *E* end section of lateral.

**Figure 10:**
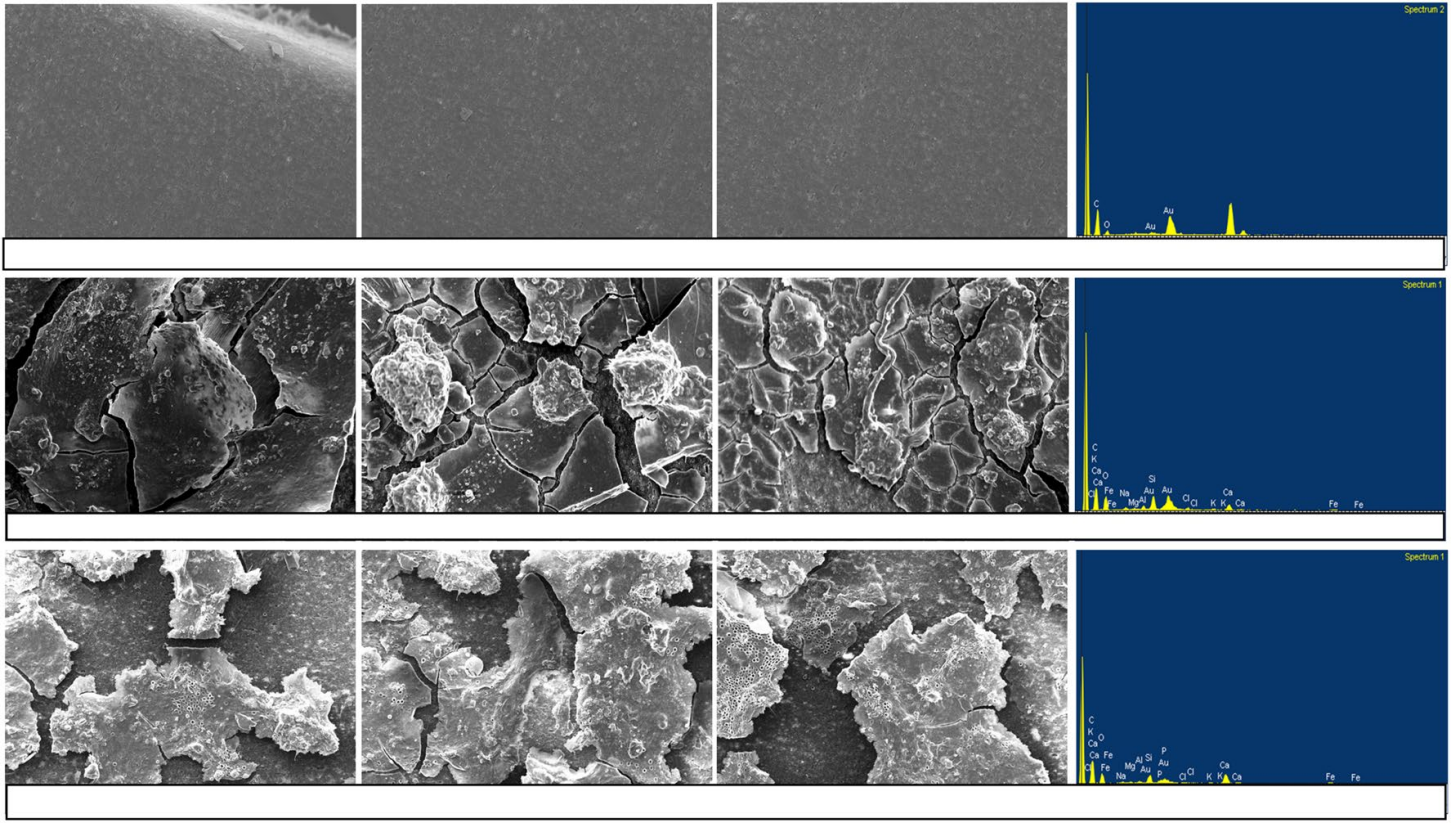
SEM–EDX for a pristine MTI lateral (top row), SEM–EDX for deposited sediment (DS) from AF effluent (second row), and SEM–EDX for (DS) from HFCW effluent (bottom row).

The bacterial colonies increased significantly over five days (Figs. 8, 9, 10). The microbial activity in deposited sediment (DS) approximately equalled that in the adhered bacterial biofilm (ABF). Hence the bacteria films formed by the DS and ABF potentially caused microbial fouling, therefore, contributing to flux decline. Microbes contribute to the formation of biofilms that resulted in stables bacterial matrices that form around the active MTI discharge nano-pores. Flux decline in membranes is due to biofilms formed after aggregated microbial communities produce a stable mechanical structure around active pores over an irrigation span^55^.

### SEM–EDX

**Figure 11:**
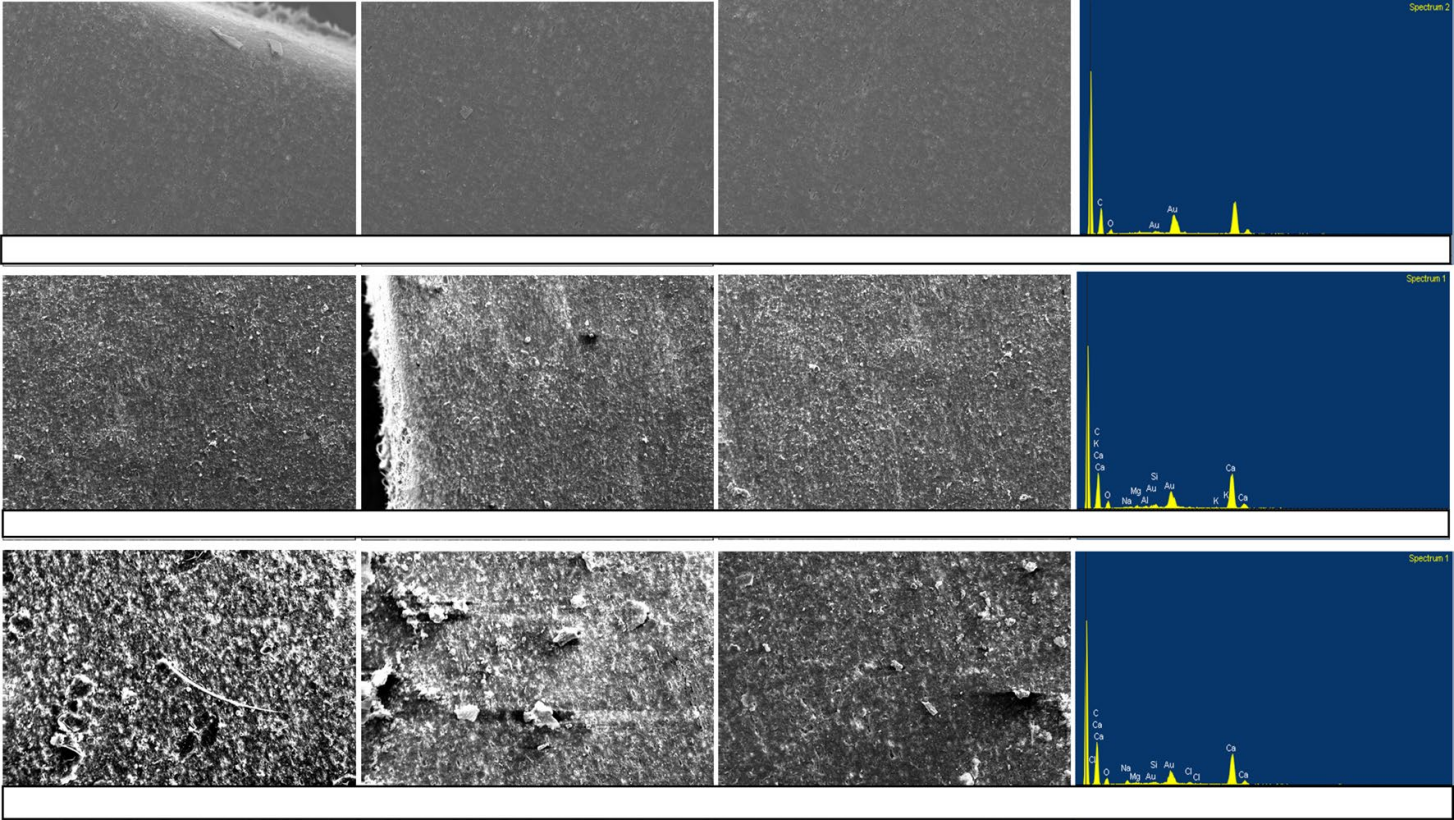
SEM–EDX for a pristine MTI lateral (top row), SEM–EDX for adhered sediment (ABF) from AF effluent (second row), and SEM–EDX for AS from HFCW effluent (bottom row).

Deposited sediment and ABF SEM–EDX images (Figs. 10, 11) revealed a dry gelatinous layer formed on the surface of the MTI. AF effluent deposited sediment covered a significant MTI surface area as compared to HFCW. ABF for both effluents formed a thin yellow layer which the imaging software could not present. The effluent produced gel layers in the MTI tubing, thus causing resistance, consequently leading to flux decline.

EDX analysis revealed that the DS from the AF and HFCW effluents contained inorganic elements. The decline in discharge for the AF and HFCW effluent could be attributed to a high TDS concentration. A one-way ANOVA test revealed there was a statistical difference between the DS and ABF from AF and that from HFCW ($p$ > 0.05) (see R-script in Supplementary Appendix I). The DS from AF had a significantly high amount of heavy metals. The pH level 7 facilitates precipitation, and long-term flocculation, which causes partial or complete pore blocking; for instance, Kanda et al.^16^ cited how inorganic compounds such as NaHCO_3_, MgSO_4_, and CaCl_2_ precipitate at pH ≥ 7 and flocculate and partially block MTI pores. The experiment by Kanda et al.^16^, a TSS concentration of 150 mg L^−1^ caused an approximately 60% decrease in MTI relative discharge over 300 h of operation whilst a TDS concentration of 2500 mg L^−1^ caused a 60% decline in relative discharge over an approximately 325 h of operation. The findings also concur with Lili et al. (2016) who posited that chemical precipitation caused emitter clogging.

## Irrigation implication

Wastewater as an alternative for freshwater irrigation has gained traction. As such, research on the effects of different effluents on irrigation technologies' performance informs operation and management (O&M) procedures. The AF and HFCW effluents significantly degraded the discharge efficiency of MTI over intermittent operation. Caution must be taken when irrigating with the respective effluents. For example, to reduce the risk of early fouling, the irrigators should perform a flushing exercise after irrigation intervals. Although this research did not perform flushing during the experiment, the exercise can potentially minimise the fouling rate. Suspended solids posed a significant risk of clogging as they were deposited on the MTI tubing surface. This negative outcome can be countered by additional filtration, although pumping systems will be required to give the irrigation water sufficient head to maintain a uniform emission. The plugging coefficients have a minute difference; however, HFCW is suitable for irrigation because of the low TSS volume. Considering that pore constriction occurs in phases, different pore locking fractals contribute differently to permeate flux reduction. Thus, phased fractal analysis will help understand the pore constriction and the consequent degree of flux decline.

## Conclusion and recommendation

The study assessed the pore blocking effects of AF and HFCW effluent. From the findings of the study, the following conclusions can be drawn:

- Effluent quality influences MTI pore blocking.
- AF has more pronounced pore-blocking as evidenced by the high degree of clogging ($DC$) and quick decline in relative discharge $\left( {q_{rel} = 0} \right)$.
- A high concentration of TSS and bacteria accelerate pore blocking. This was evidenced by a high $DC$ and quick decline in $q_{rel}$ (AF, $q_{rel} = 0$ at $t$ = 57 h and HFCW, $q_{rel} = 0$ at $t$ = 60 h)
- MTI permeate flux decline follows a typical time vs flux curve
- For both AF and HFCW effluents, MTI plugging coefficients were 0.07 and 0.05, respectively.
- The SEM revealed cake formation layer under AF effluent that covered a significant surface area, thus reducing active MTI pores for discharge.

Additional filtering of irrigation water can potentially mitigate the effects of MTI plugging by wastewater effluent. Future research is required to assess the actual performance of a buried MTI lateral under wastewater irrigation with filtration. Also, the researchers recommend the experiment be run under continuous irrigation scenarios. It is worth noting that MTI pore locking happens in phases depending on the characteristic effluent. Stage by stage fractal analysis will help determine the build-up to the eventual pore-blocking coefficients. Thus, a future study is recommended to determine the characteristic fractal formation of the different effluents and their effects on MTI fouling.

## Supplementary Information

Supplementary Information 1.

Supplementary Information 2.

Supplementary Information 3.

Supplementary Information 4.

## Data Availability

Supporting has been made available to Editorial Board Members and referees at the time of submission for the purposes of evaluating the manuscript (see Supplementary Information).
