# Supplementary material for: Moistube irrigation fouling due to anaerobic filtered effluent (AF) and horizontal flow constructed wetland (HFCW) effluent
Source: Sci Rep. 2021 Mar 29;11:7124. doi: 10.1038/s41598-021-86737-7 (PMC8007708; doi:10.1038/s41598-021-86737-7)

**Moistube Irrigation Fouling Due to Anaerobic Filtered Effluent (AF) and Horizontal Flow Constructed Wetland (HFCW) Effluent**

T L Dirwai^1,2^*, A Senzanje^1^, T Mabhaudhi^3,4^, C A. Buckley^5^

^1^*School of Engineering, University of KwaZulu-Natal, Agricultural Engineering Department, P.Bag X01, Pietermaritzburg, South Africa*

^2^*VarMac Consulting Engineers, Scottsville, Pietermaritzburg, 3209, South Africa*

^3^*Center for Transformative Agricultural and Food Systems, School of Agricultural, Earth and Environmental Sciences, University of KwaZulu-Natal, Pietermaritzburg, P.Bag X01, South Africa*

^4^*Center for Water Resources Research, School of Agricultural, Earth and Environmental Sciences, University of KwaZulu-Natal, P. Bag X01, Pietermaritzburg 3209, South Africa*

^5^ *Water, Sanitation & Hygiene Research & Development Centre, Chemical Engineering, School of Engineering, University of KwaZulu-Natal, Durban 4041, South Africa*

Correspondence to: TLD (tldirwai@gmail.com)

**Appendix I**


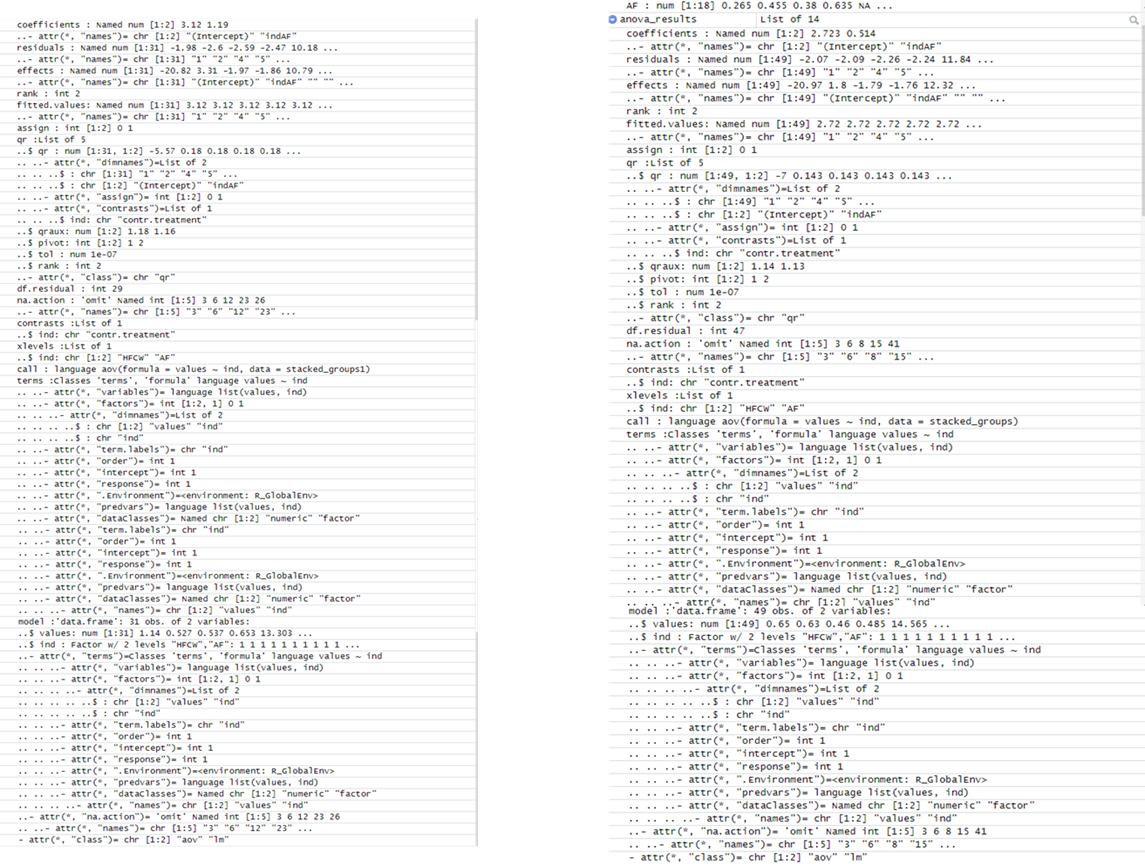

Supplement: Supplementary file 4 — Supplementary Information 4. [file 41598_2021_86737_MOESM4_ESM.docx]
